# Essential Oil Nanoemulsion Hydrogel with Anti-Biofilm Activity for the Treatment of Infected Wounds

**DOI:** 10.3390/polym15061376

**Published:** 2023-03-09

**Authors:** Kun Cai, Yang Liu, Yan Yue, Yuancheng Liu, Fengbiao Guo

**Affiliations:** Guangdong Provincial Key Laboratory of Marine Biotechnology, Department of Biology, College of Science, Shantou University, Shantou 515063, China

**Keywords:** eucalyptus essential oil, nanoemulsion, hydrogel, antibacterial, anti-biofilm, infected wounds

## Abstract

The formation of a bacterial biofilm on an infected wound can impede drug penetration and greatly thwart the healing process. Thus, it is essential to develop a wound dressing that can inhibit the growth of and remove biofilms, facilitating the healing of infected wounds. In this study, optimized eucalyptus essential oil nanoemulsions (EEO NEs) were prepared from eucalyptus essential oil, Tween 80, anhydrous ethanol, and water. Afterward, they were combined with a hydrogel matrix physically cross-linked with Carbomer 940 (CBM) and carboxymethyl chitosan (CMC) to prepare eucalyptus essential oil nanoemulsion hydrogels (CBM/CMC/EEO NE). The physical-chemical properties, in vitro bacterial inhibition, and biocompatibility of EEO NE and CBM/CMC/EEO NE were extensively investigated and the infected wound models were proposed to validate the in vivo therapeutic efficacy of CBM/CMC/EEO NE. The results showed that the average particle size of EEO NE was 15.34 ± 3.77 nm with PDI ˂ 0.2, the minimum inhibitory concentration (MIC) of EEO NE was 15 mg/mL, and the minimum bactericidal concentration (MBC) against *S. aureus* was 25 mg/mL. The inhibition and clearance of EEO NE against *S. aureus* biofilm at 2×MIC concentrations were 77.530 ± 7.292% and 60.700 ± 3.341%, respectively, demonstrating high anti-biofilm activity in vitro. CBM/CMC/EEO NE exhibited good rheology, water retention, porosity, water vapor permeability, and biocompatibility, meeting the requirements for trauma dressings. In vivo experiments revealed that CBM/CMC/EEO NE effectively promoted wound healing, reduced the bacterial load of wounds, and accelerated the recovery of epidermal and dermal tissue cells. Moreover, CBM/CMC/EEO NE significantly down-regulated the expression of two inflammatory factors, IL-6 and TNF-α, and up-regulated three growth-promoting factors, TGF-β1, VEGF, and EGF. Thus, the CBM/CMC/EEO NE hydrogel effectively treated wounds infected with *S. aureus*, enhancing the healing process. It is expected to be a new clinical alternative for healing infected wounds in the future.

## 1. Introduction

Wound infection represents a severe complication of skin injuries and is usually formed when pathogenic bacteria, such as *S. aureus,* attach to a wound and multiply on it [1,2]. Wound infection can impede the normal healing process, greatly prolonging healing time. Lacking effective treatment measures, wound infection can be lethal for patients [3]. It has been previously shown that biofilms are present in over 80% of infected wounds [4,5,6]. Biofilms act as a physical barrier and prevent the penetration of immune cells and therapeutic agents. This physical barrier can make wound healing more difficult, increase the consumption of medical resources, and intensify the suffering experience of patients [7,8,9]. Antibiotic treatment is still one of the most common methods for treating infected wounds in clinical practice. However, bacteria wrapped in biofilms are about 1000 times more antibiotic-resistant than planktonic bacteria, diminishing the overall effects of antimicrobials and challenging their widespread use for treating infectious wounds [10]. Moreover, the overuse of antimicrobials can lead to severe cytotoxicity and bacterial resistance, making treating infected wounds even more difficult and creating a vicious circle [9,11]. It is, therefore, essential to develop a wound dressing that effectively inhibits biofilm formation and growth to treat bacterially infected wounds.

Researchers have developed different types of antimicrobial agents for treating infections of traumatic surfaces against bacteria and biofilms. Commonly used antimicrobial agents are classified into three categories based on the type of materials used: inorganic, biological, and organic antimicrobial agents [12]. Inorganic antimicrobial agents can be divided into two categories: metal-based and carbon-based. Typical materials include silver nanoparticles, titanium dioxide nanoparticles, graphene, etc. However, the use of these inorganic antimicrobial agent materials is accompanied by high manufacturing costs, short and insufficient antimicrobial capacity, and an unknown impact on human health and the environment [12,13,14,15,16,17,18]. Biological antibacterial agents, such as enzymes, peptides, and bacteriophages, originate from living organisms. Compared to inorganic antibacterial agents, biological antibacterial agents can be combined with different substrates using various surface modification reactions and intermolecular interactions. So, biological antibacterial agents have a wider range of applications. However, the use of biological antibacterial agents is expensive and limited by the longevity of their biological sources and environmental factors [12,19,20,21]. Organic antibacterial agents can be divided into synthetic and naturally derived materials. Synthetic organic antibacterial agents include common organic antimicrobials, such as penicillin and amoxicillin, which are widely used but prone to developing resistance in pathogenic bacteria [11,12,22]. Among naturally derived organic antimicrobial agents, essential oils have gained widespread interest due to their low toxicity, broad-spectrum antibacterial activity, multiple pharmacological activities, and relatively low cost [23,24,25]. Our laboratory has screened an essential oil extracted from the eucalyptus tree—eucalyptus essential oil (EEO)—by screening the antibacterial activity in vitro in the early stage. The essential oil showed a strong in vitro inhibitory effect on *S. aureus*. However, the volatile and slightly pungent smell of essential oil affects its long-term antibacterial performance, limiting its further application and development [26]. Therefore, researchers have developed different carrier systems to load and deliver essential oils, including biopolymers, surfactants, and lotions [27,28]. Among them, nanoemulsions represent a lotion-based carrier system that has attracted increasing attention because they can effectively enhance the physical stability and antibacterial activity of essential oils [29]. The preparation process of nanoemulsions is simpler than that of other carrier systems and the cost is relatively low. In addition, the composition of nanoemulsions can be tailored using a wide range of sources and is not limited to the synthesis of surfactants [30,31,32,33,34,35]. Therefore, EEO can be encapsulated in a nanoemulsion to enhance its stability and antibacterial activity.

To address the recovery of infected wounds more effectively, researchers usually combine antibacterial agents with various matrix materials to prepare wound dressings with different properties. Common matrix materials mainly include hydrogels, nanofibers, films, and nanomaterials. Among these, hydrogels are a popular choice for wound dressing due to their biocompatibility, flexibility, and high-water content [36,37]. S. A. Razack et al. [38] prepared a trauma dressing by combining an oregano essential oil nanoemulsion with a hydrogel matrix prepared from chitosan and gelatin. The dressing exhibited good antibacterial properties and effectively promoted wound recovery. In addition, nanoparticle-loaded hydrogels have a wide range of other applications in the biomedical field. They can be used as a reaction platform for qPCR [39], or as a method for continuous, extreme lubrication of hydrogels in applications ranging from tissue engineering to clinical diagnostics [40]. They can also be used as drug carriers for the treatment of bone infections caused by bacteria [41].

Our laboratory has developed a hydrogel based on the physical cross-linking of carboxymethyl chitosan (CMC) and Carbomer 940 (CBM). It exhibits good water retention and water vapor permeability properties, and provides a stable, moist, and breathable environment for wounds as required, according to the theory of moist wound healing. Moreover, this hydrogel can also be used as a matrix material combined with various active substances to meet different wound repair requirements [26].

In this study, EEO was encapsulated in a nanoemulsion carrier and creatively compounded with a CBM/CMC matrix. A trauma dressing with essential oil nanoemulsions (EEO NEs) as an antimicrobial agent was developed for the treatment of infected wounds. The physicochemical properties, in vitro antibacterial and antibiofilm activity, biocompatibility, and in vivo repair capacity of EEO NEs and hydrogels were examined to evaluate their potential in the treatment of infected wounds and to provide an idea for further research of new materials for the treatment of infected wounds.

## 2. Materials and Methods

### 2.1. Materials

Eucalyptus essential oil was purchased from ZRZR biotechnology Co. (Guangzhou, China). carboxymethyl chitosan (carboxylation degrees ≥ 80%) and Carbomer 940 (CAS. NO. 54182-57-9) were supplied by Yuanye biotechnology Co. (Shanghai, China). Triethanolamine (CP, ≥99.0%) was procured from Aladdin Reagent Co. (Shanghai, China). Tween 80 (Oleic acid approx.70%), Luria-Bertani (LB) Broth, a CCK-8 detection kit, and an ELISA kit were supplied by Solarbio (Beijing, China). Chloral hydrate was obtained from Macklin Biochemical Co. (Shanghai, China). All other reagents used were of analytical grade (AR, ≥99.7%).

### 2.2. Preparation of EEO NEs and EEO NE Hydrogels

Nanoemulsions were prepared via low-energy emulsification [42] using EEO, Tween 80, anhydrous ethanol, and water as raw materials. Tween 80 was the surfactant, and anhydrous ethanol was the co-surfactant, and they were used in a 2:1 mass ratio to prepare the surfactant mix. A total of 3 g of EEO was added to 7 g of the surfactant blend and stirred, followed by the dropwise addition of 25 mL of deionized water using a burette to obtain a nanoemulsion master batch. Deionized water was dropwise added to the nanoemulsion master batch until the total nanoemulsion system reached 100 g. Then, the eucalyptus essential oil nanoemulsion with an essential oil content of 3% (g/g) was prepared.

Preparation of hydrogel was conducted with reference to Wang’s method [26]. A total of 0.5 g of CBM was dissolved in 50 mL of deionized water and left to swell overnight at room temperature. 5 mL of a 10% CMC solution was added and stirred well; then, triethanolamine was added dropwise to adjust the pH to neutral. At this point, a blank hydrogel—CBM/CMC was obtained when water was added to the total system up to a mass of 100 g and stirred well; when the nanoemulsion master batch prepared above was added and then deionized, water was poured to a total gel mass of 100 g, so the eucalyptus essential oil nanoemulsion hydrogels (CBM/CMC/EEO NE) was obtained.

### 2.3. Characterization of EEO NE and CBM/CMC/EEO NE

#### 2.3.1. Basic Properties and the Particle Size Distribution of EEO NE

The basic criteria for characterizing EEO NEs were determined by pure optical observation: 1. Is the emulsion clear and transparent? 2. Does the emulsion exhibit a blue or light blue opalescence? 3. Is there a Tyndall effect when parallel light is applied? The pH of the EEO NE was determined using a pH meter. The water-soluble dye methylene blue and the oil-soluble dye Sudan red III were added dropwise to the emulsion at the same time, and the type of emulsion was determined by the rate of diffusion of the two dyes in the emulsion.

The properties of nanoemulsions were determined with reference to the method of Hou et al. [33]. The prepared EEO NE was diluted 50 times with distilled water to prepare the sample for testing, and the particle size, particle size distribution, and polydispersity index were determined using a nanoparticle size analyzer (ZETASIZER NANO ZS9 Malvern, Malvern, UK).

#### 2.3.2. Rheological Measurements of CBM/CMC/EEO NE

The rheological properties of the hydrogels were determined with reference to the method of Wang et al. [26]. In this experiment, a TA-AR2000ex rheometer was used to measure the energy storage modulus (G’) and loss modulus (G’’) of CBM/CMC and CBM/CMC/EEO NE composite hydrogel samples using dynamic frequency scanning (0.01% strain). These two parameters reflect the elasticity and viscosity of the object, respectively. The measurements were performed at 25 °C and in the scanning range of 0.1–10 Hz.

#### 2.3.3. Water Loss Rate of CBM/CMC/EEO NE

The assay was performed with reference to the method of Wang et al. [26]. All samples were measured in triplicate. A total of 1 g of each CBM/CMC and CBM/CMC/EEO NE composite hydrogel sample was weighed in 2 mL EP tubes, and their initial mass was accurately determined (M_0_). The hydrogel samples were then placed in a silica gel desiccator for 36 h at 37 °C and weighed accurately at regular intervals (M_a_). The final mass was measured after 36 h (M_b_). The water loss rate (%) was calculated using Equation (1):

Water loss rate (%) = (M_a_ − M_0_)/(M_0_ − M_b_) × 100%. (1)

#### 2.3.4. Porosity of CBM/CMC/EEO NE

An assay was performed with reference to the method of Lin et al. [43]. All samples were measured in triplicate. Appropriate amounts of CBM/CMC and CBM/CMC/EEO NE lyophilized samples were measured and denoted as M_0_. Furthermore, we measured their volume, denoted as V_0_. The samples were soaked in 50 mL of ethanol, and the mass of an empty bottle (M_1_) was measured before being put in a sonicator to remove air bubbles for 2 min so that the liquid filled the pores of the gel. Next, we pour out the excess ethanol and measure the total mass (M) of the remaining substance. The experiment was repeated three times in parallel to calculate the porosity using Equation (2), where the ethanol density was 0.789 g/cm^3^:

Porosity (%) = (M − M_1_ − M_0_)/0.789/V_0_ × 100%. (2)

#### 2.3.5. Water Vapor Transmission Rate (WVTR) Test for CBM/CMC/EEO NE

An assay was performed with reference to the method of Wang et al. [26]. All samples were measured in triplicate. A glass vial 18 mm in diameter was filled with 10 mL of deionized water, and the mouth of the vial was covered with a layer of gauze. A total of 0.5 g of the measured CBM/CMC and CBM/CMC/EEO NE composite hydrogels was weighed and evenly covered with gauze, and the mouth of the vial was sealed. Their initial mass was accurately weighed (M_0_). The hydrogel-covered bottles were placed in a silica gel desiccator and removed at 37 °C for 12, 24, 36, and 48 h to accurately measure their mass (Ma). The WVTR (mg/m^2^/day) was calculated as follows (3):

WVTR (mg/m^2^/day) = (M_0_ − M_a_)/10^6^ A × T, (3)

where A is the area of the bottle opening and T is the number of days.

### 2.4. In Vitro Antibacterial Activity

#### 2.4.1. Determination of the Minimum Inhibitory Concentration (MIC) and Minimum Bactericidal Concentration (MBC) of EEO NE

The MIC and MBC of EEO NE against *S. aureus* were determined with minor modifications of existing experimental methods [44,45]. *S. aureus* was incubated in the LB medium up to the logarithmic growth phase, and the concentration was adjusted to 1 × 10^6^ CFU/mL via absorbance testing. The diluted bacterial solution was added to a 96-well plate with 100 μL per well, and then 100 μL of the gradiently diluted EEO NE was added to each well to ensure that the concentration of EEO NE in wells ranged from 0.05 to 50 mg/mL. After incubation at 37 °C for 24 h, the minimum sample concentration without significant turbidity or precipitation in the wells was the MIC. Afterward, 100 μL of the culture solution was taken from the wells without visible bacterial growth, coated in LB solid medium plates, and incubated at 37 °C for 24 h. The growth of colonies in the plates was monitored, and the minimum sample concentration represented by the plate without visible bacterial growth was the MBC. Each experiment was repeated at least twice.

#### 2.4.2. Inhibitory Activity Assay of EEO NE and CBM/CMC/EEO NE against Suspended *S. aureus*

The antimicrobial activities of EEO NE and CBM/CMC/EEO NE were determined against *S. aureus* bacterial strains using the turbidimetric analysis method [26,46]. *S. aureus* was incubated in the LB medium to the logarithmic growth phase, and the concentration was adjusted to 1 × 10^6^ CFU/mL via absorbance testing. A total of 100 μL of the negative control (sterile water), positive control (50 μg/mL of gentamicin sulfate solution), EEO NE, and CBM/CMC/EEO NE were added to each well of a 96-well plate, followed by the addition of 100 μL of the bacterial solution to each well and incubated at 37 °C for 48 h, during which 100 μL of the culture solution was taken at 12 h intervals, and the absorbance values were measured at 595 nm using an enzyme marker (ELX-80, Burton Instruments, Rockville Maryland, USA) with at least 3 parallels in each group. The antibacterial rate (%) was calculated as follows (4):

Antibacterial rate (%) = (OD_0_ − OD_1_)/OD_0_ × 100%, (4)

where OD_0_ indicates the absorbance value of the negative control well and OD_1_ indicates the absorbance value of the positive control/sample well.

#### 2.4.3. Inhibitory Activity of EEO NE on the *S. aureus* Biofilm Formation

The activity of EEO in inhibiting biofilm formation was determined using crystalline violet staining [47]. A 96-well plate was incubated at a concentration of 1 × 10^6^ CFU/mL in 100 μL per well, followed by the addition of 100 μL of negative control (sterile water), positive control (50 μg/mL of gentamicin sulfate solution), and EEO NE (0.5 × MIC, 1 × MIC, and 2 × MIC gradients) to the wells. It was incubated at 37 °C for 24 h, followed by aspiration of the bacterial solution. After the incubation, 200 μL of 1% crystalline violet solution was added to each well for 30 min; the staining solution was aspirated and rinsed with PBS, then 200 μL of 33% acetic acid solution was added to each well for 30 min, after which 100 μL of the solution was taken, and the absorbance value was measured at 595 nm, with at least 3 parallels in each group. The biofilm eradication rate was calculated according to formula (5) to evaluate the inhibitory activity on biofilm formation:

Biofilm eradication rate (%) = (OD_0_ − OD_1_)/OD_0_ × 100%, (5)

where OD_0_ indicates the absorbance value of the negative control well and OD_1_ indicates the absorbance value of the positive control/sample well.

#### 2.4.4. Determination of the Scavenging Activity of EEO NE on *S. aureus* Biofilms

A 96-well plate was incubated at 37 °C for 48 h at a concentration of 1 × 10^6^ CFU/mL in 200 μL per well to form a mature biofilm. After aspiration and rinsing with PBS, 200 μL of negative control (sterile water), positive control (50 μg/mL of gentamicin sulfate solution), and EEO NE (0.5 × MIC, 1 × MIC, and 2 × MIC gradients) were added to each well and incubated at 37 °C for 4 h. The biofilm clearance activity was then evaluated as the biofilm elimination rate, as described in Section 2.4.3.

### 2.5. In Vitro Biocompatibility Tests

#### 2.5.1. In Vitro Cytotoxicity Assay

An assay was performed with reference to the method of Wang et al. [26]. A total of 0.1 g of CBM/CMC and CBM/CMC/EEO NE hydrogels were immersed in 1 mL of the DMEM medium and soaked at 37 °C for 24 h to make a master batch of 0.1 g/mL hydrogel extract. The mother liquor was then diluted with the DMEM medium to 1, 10, 100, 1000, and 10,000 μg/mL of gel extracts, which were subsequently UV-irradiated for 2 h and then decontaminated with a 0.22-μm filter membrane and set aside.

L929 cells were inoculated in 96-well plates at a density of 1 × 10^5^ per well, incubated at 37 °C for 24 h until the cells were plastered, then the medium was aspirated, the blank group was replaced with the fresh DMEM medium, and the gel group was replaced with each gel extract and incubated again for 24 h. Then, the cells were assayed according to the CCK-8 kit, and the cell survival rate was calculated. All samples were measured in triplicate.

#### 2.5.2. Blood Compatibility Test

10,000 μg/mL of CBM/CMC and CBM/CMC/EEO NE hydrogel extracts were prepared according to 2.5.1. A total of 100 μL of a 4% chicken blood erythrocyte suspension was added to 1 mL of each saline (negative control), deionized water (positive control), and the above gel extracts in a centrifuge tube and incubated at 37 °C for 1 h. The supernatant was centrifuged at 8000 r/min for 5 min, and the absorbance was measured at 545 nm, with at least 3 parallels in each group. The hemolysis rate (%) was calculated according to the following formula:

Hemolysis rate (%) = (OD_S_ − OD_−_)/(OD_+_ − OD_−_) × 100%, (6)

where OD_S_ is the OD value of the sample supernatant and OD_+_ and OD_−_ are the OD values of the negative control and positive control, respectively.

### 2.6. In Vivo Wound Healing Evaluation

#### 2.6.1. Infected Trauma Molding

The infected trauma model was constructed with reference to the method of Zhang et al. [4]. Age-appropriate male mice weighing 30–40 g were divided into negative, positive, and experimental groups. The mice were anesthetized before the experiment, and then the hair on their back skin was removed using an animal electric shaver. The surgical area on the back skin was disinfected with 75% alcohol, and an 8 mm circular wound was created on each mouse. 100 μL of a suspension of *S. aureus* at a concentration of 1 × 10^7^ was added to the wound and left for 15 min to allow bacteria to colonize the wound and construct a wound with chronic *S. aureus* infection.

#### 2.6.2. Analysis of Wound Healing Rates

Daily administration was started 24 h after molding with no treatment in the negative group, 1% erythromycin ointment in the positive group, and the CBM/CMC/EEO NE composite hydrogel in the experimental group. On days 1, 4, 8, 12, and 16, the area of each wound treated in the different groups was measured and calculated using ImageJ software, with at least 6 parallels in each group. The wound healing rate (%) was calculated according to the following formula:

Wound healing rate (%) = (S_1_ − S_n_)/S_1_ × 100%, (7)

where S_1_ is the area of the trauma on the first day of molding and S_n_ is the area of the trauma on the day n of molding.

#### 2.6.3. Trauma Tissue Sampling

Eight mice from each group were executed on days 1, 4, 8, 12, and 16, respectively, and the entire skin wound was cut off about 1 cm along the outer edge of the wound and washed with cold saline. The tissue was divided into three parts, one fixed in 10% formalin for histopathological sectioning, one frozen at −80 °C for cytokine detection, and one for bacterial load detection.

#### 2.6.4. Trauma Bacterial Load Testing

The tissue was weighed and ground into a 0.1 g/mL tissue homogenate with saline, diluted in a gradient, spread on LB solid medium plates, and incubated for 24 h at 37 °C, and colonies were counted to calculate the bacterial load in the tissue.

#### 2.6.5. Histological Testing

The fixed skin tissues were trimmed, washed, and dehydrated, and then made transparent in xylene, paraffin-embedded, and sectioned at 4–6 μm. The obtained tissue sections were dewaxed with lignin and stained with Hematoxylin-Eosin (H&E) and Masson’s Trichrome Staining, respectively, and the changes in tissue morphology were examined using a light microscope (AXIO IMAGER Z1 Zeiss, Oberkochen, Germany).

#### 2.6.6. Tissue Cytokine Assays

The trauma skin was first mixed in a ratio of 1:19 with saline at low temperature and put into a glass homogenizer to make a 5% tissue homogenate, centrifuged at 2000~3000 r/min for 20 min, and the supernatant, i.e., the sample to be tested, was taken. Then, the levels of interleukin-6 (IL-6), tumor necrosis factor α (TNF-α), transforming growth factor β1 (TGF-β1), vascular endothelial growth factor (VEGF), and epidermal growth factor (EGF) in the trauma tissue were measured using an ELISA kit.

### 2.7. Statistical Analysis

All measurements were performed in triplicate and were reported as calculated means and standard deviations (mean ± SD). Statistical analysis (ANOVA analysis and Dunnett’s multiple comparison Test) was performed using SPSS 22.0 software, professional edition. The level of significance was determined as *p* < 0.05.

## 3. Results and Discussion

### 3.1. Characteristics of EEO NE and CBM/CMC/EEO NE

**Figure 1:**
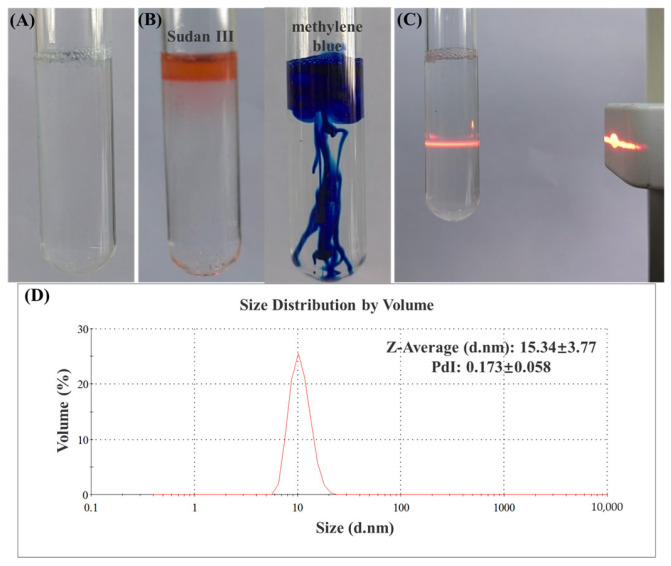
(**A**) External view of EEO NE. (**B**) Identification of the emulsion type with Sudan III as the red dye and methylene blue as the blue dye. (**C**) Parallel light incident on EEO NE to form a stable light path. (**D**) Particle size, distribution, and polydispersity index results of EEO NE (mean ± SD).

Optical observation provides a direct insight into the relevant properties of nanoemulsions. As shown in Figure 1, the resulting EEO NEs are clear and transparent, exhibiting a light blue color and showing a Tyndall effect when parallel light is directed at them. These phenomena are consistent with the basic criteria for nanoemulsions. When both Sudan III and methylene blue dyes are added, water-soluble methylene blue dye diffuses more rapidly in the emulsion, indicating that the emulsion type is oil-in-water. The average particle size of the prepared EEO NE is 15.34 ± 3.77 nm, and the PDI value is 0.173 ± 0.058, i.e., less than 0.2, indicating that the emulsion exhibits a uniform particle size distribution. The EEO NE shows a neutral pH value of 7, which is suitable for treating skin wounds.

**Figure 2:**
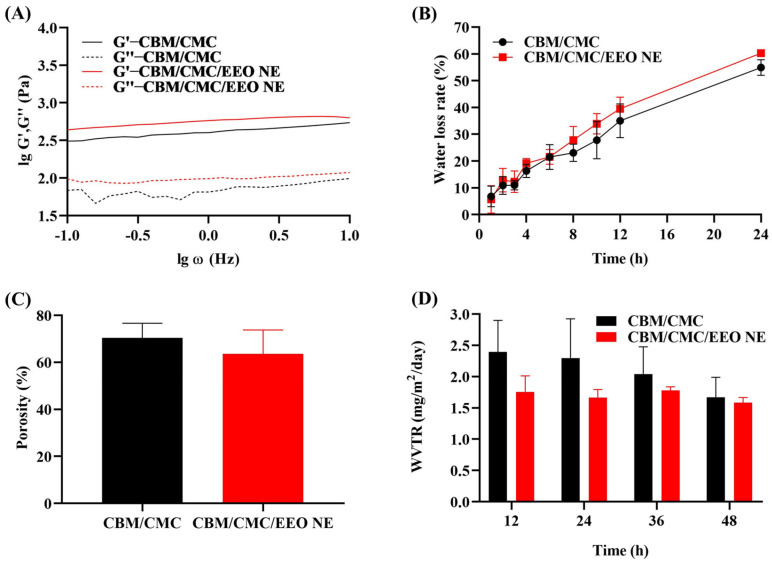
Properties of CBM/CMC/EEO NE. (**A**) Rheological test, G’—energy storage modulus, G’’—loss modulus. (**B**) Water loss rate within 24h. (**C**) Porosity. (**D**) Water vapor transmission rate within 48 h.

The examination of the rheological properties of hydrogels reflects the changes in their viscosity and elasticity. As shown in Figure 2A, the elastic modulus of both CBM/CMC and CBM/CMC/EEO NE hydrogels is slightly higher than the loss modulus, indicating that both hydrogels exhibit solid state properties. Combined with the fact that both moduli of the hydrogels are slightly frequency dependent, we assume that both hydrogels exhibit a weak gelation behavior. In general, the storage modulus G’ corresponds to the elasticity of the measured fluid, while the loss modulus G” corresponds to the fluid’s viscosity. Figure 2A shows that the elasticity and viscosity of the hydrogel with added EEO NE are improved compared to the sample without EEO NE, and the performance improvement can be better applied to improve wound repair.

The lower the water loss rate, the better the water retention properties of the hydrogel. Water retention is an important indicator of the properties of hydrogel dressings, as it creates a moist environment that facilitates the growth, migration, and differentiation of trauma cells, thus promoting wound healing. As shown in Figure 2B, the water loss rate of CBM/CMC and CBM/CMC/EEO NE hydrogels increases over 24 h, reaching 54.942 ± 2.962% and 60.268 ± 1.053%, respectively, at the 24th hour, wherein CBM/CMC/EEO NE exhibits a slightly higher value than CBM/CMC. It is speculated that the addition of EEO NE may have reduced the ability of the hydrogel to bind water, resulting in higher water loss. However, both hydrogels exhibit a long-lasting water-retention capacity, which is conducive to maintaining a moist environment on wounds, promoting collagen production and autoimmune repair.

It is important that the moist environment created by a hydrogel dressing has an appropriate water vapor transmission rate, since only then it maintains the moisture content around the wound and promotes cell proliferation and wound healing [48,49]. When the water vapor transmission rate is too high, it tends to dry out the wound, which is not conducive to cell growth and the maintenance of a moist environment; furthermore, when the water vapor transmission rate is insufficient, it may allow exudate to accumulate on the wound, resulting in slow wound healing. As shown in Figure 2D, the water vapor transmission rate of CBM/CMC/EEO NE is lower than that of CBM/CMC during the 48 h test, but the change in the water vapor transmission rate is more stable than that of CBM/CMC, indicating that CBM/CMC/EEO NE has a stable water vapor transmission rate and can maintain a moist and permeable environment for the wound surface. The water vapor transmission rate is closely related to porosity. As shown in Figure 2C, the porosity of CBM/CMC and CBM/CMC/EEO NE is 70.430 ± 6.193% and 63.586 ± 10.166%, respectively. Lower porosity results in a lower water vapor transmission rate, i.e., the water vapor transmission rate of CBM/CMC/EEO NE is lower than that of CBM/CMC.

### 3.2. In Vitro Antibacterial Activity

The minimum inhibitory concentration and the minimum bactericidal concentration of EEO NE were determined by the standard broth dilution method. The experimental results show that the minimum use concentration of EEO NE without visible bacterial growth is 15 mg/mL; i.e., the MIC value of EEO NE is 15 mg/mL. By observing the colony production of the well plate culture on the plate, it is determined that the minimum use concentration of EEO NE without visible bacterial growth is 25 mg/mL; i.e., the MBC value of EEO NE is 25 mg/mL.

**Figure 3:**
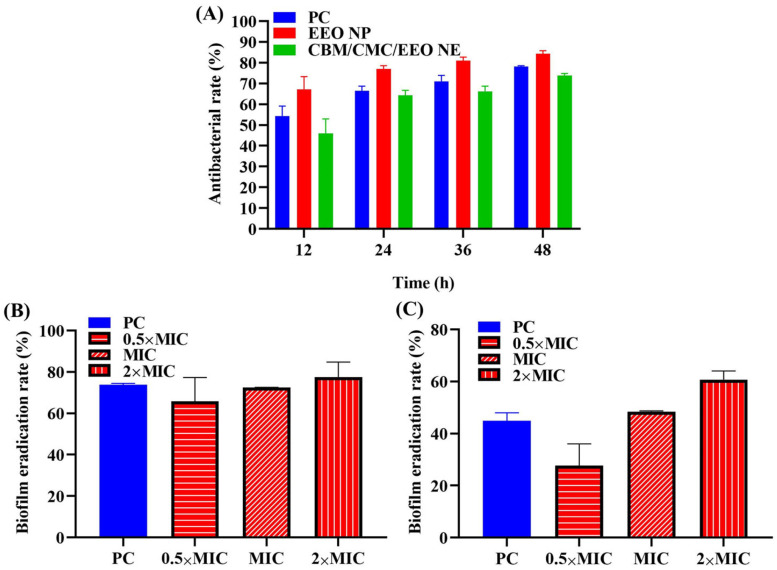
In vitro antibacterial activity. (**A**) Inhibitory activity of EEO NE and CBM/CMC/EEO NE against suspended *S. aureus* within 48 h. (**B**) Inhibitory activity of EEO NE against *S. aureus* biofilm formation at different concentrations (0.5×, 1× and 2 × MIC). (**C**) Removal activity of EEO NE against *S. aureus* biofilm at different concentrations (0.5×, 1×, and 2 × MIC).

The inhibition activities of EEO NE and CBM/CMC/EEO NE against suspended *S. aureus* were measured by absorbance value assay at 48 h. As shown in Figure 3A, the inhibition rates of EEO NE and CBM/CMC/EEO NE are higher than 50% from 24 h onward, and they increase in the subsequent time, indicating that both EEO NE and CBM/CMC/EEO NE exhibit good long-lasting inhibition activity against the suspended *S. aureus*. The inhibition rate of EEO NE is higher than that of the treatment group of the gentamicin sulfate solution positive control within 48 h, indicating that the inhibition activity of the nano-emulsified EEO is comparable to or even slightly higher than that of the usual antimicrobials. This suggests that EEO NE has the potential to replace antimicrobials as a bacteriostatic substance. When EEO NE is combined with the hydrogel matrix to form the CBM/CMC/EEO NE hydrogel, its bacterial inhibition activity is reduced compared to EEO NE within 48 h, presumably due to the reduced release of EEO NE in the bacterial solution after combining it with the gel matrix. However, the inhibition rate after 24 h is greater than 50%, still exhibiting excellent bacterial inhibition ability.

In the in vitro anti-biofilm formation and biofilm clearance assay of EEO NE (shown in Figure 3B,C), the inhibitory effect of EEO NE on the biofilm formation and clearance of *S. aureus* is dose-dependent. The 2×MIC concentration of EEO NE shows the highest inhibitions of the biofilm formation and clearance of *S. aureus*, 77.530 ± 7.292% and 60.700 ± 3.341%, respectively, which are higher than the positive control of 73.775 ± 0.720% and 44.900 ± 3.035%. This indicates that EEO NE has a superior ability to inhibit biofilm formation and remove mature biofilms compared to common antimicrobials. It further demonstrates the potential of EEO NE as a biofilm-inhibiting active substance that can be added to hydrogels for the treatment of infected chronic wounds.

### 3.3. In Vitro Biocompatibility

**Figure 4:**
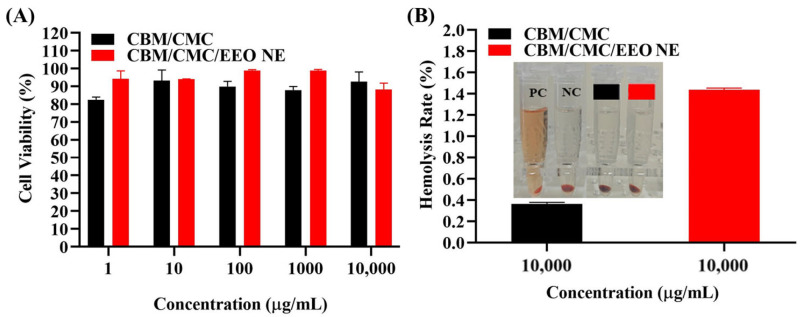
In vitro biocompatibility. (**A**) Cytotoxicity analysis of different concentrations (1–10,000 μg/mL) of CBM/CMC and CBM/CMC/EEO NE hydrogel extracts on mouse fibroblasts L929. (**B**) 10,000 μg/mL CBM/CMC and CBM/CMC/EEO NE hydrogel extracts for hemolysis of chicken blood erythrocytes.

The biocompatibility of CBM/CMC/EEO NE needs to be evaluated in vitro by cytotoxicity assay and hemolysis assay before in vivo experiments. As shown in Figure 4A, the survival rates of L929 cells under separate cultures of 1, 10, 100, 1000, and 10,000 μg/mL CBM/CMC/EEO NE hydrogel extracts range from 88.235 to 98.799%, and all of these are higher than 85%. This indicates that the hydrogel is not cytotoxic at concentrations equal to or below 10,000 μg/mL and can be used as a safe trauma dressing.

In the hemolysis experiment (Figure 4B), hemolysis appeared in the positive control group, while the hydrogel group had no hemolysis. The calculated hemolysis rates of the CBM/CMCP and CBM/CMC/EEO NE groups are both less than 5%, meeting the clinical requirements for the hemolysis rate of biological materials. It can be concluded that the CBM/CMC/EEO NE hydrogel has good hemocompatibility.

### 3.4. In Vivo Experiments

#### 3.4.1. Analysis of Wound Healing

**Figure 5:**
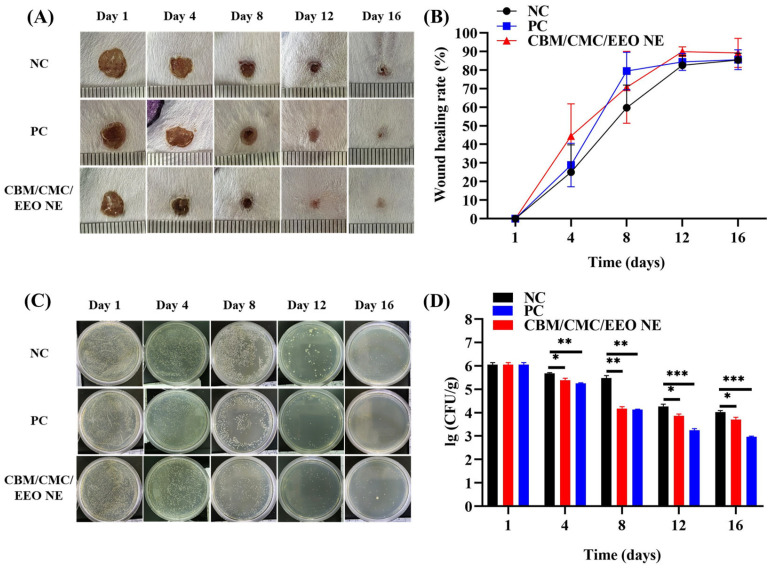
Wound healing and bacterial load analysis of infected trauma within 16 days. (**A**) Appearance of infected wound healing in mice. (**B**) Wound healing rate curve. (**C**) Plate count assay of bacterial content of infected trauma tissue in mice. (**D**) Wound tissue bacterial load analysis. (n = 3, * *p* < 0.05, ** *p* < 0.01 and *** *p* < 0.001, vs. NC).

The therapeutic effect of the CBM/CMC/EEO NE hydrogel on the wounds infected with *S. aureus* was evaluated by recording the change in the wound area in mice. As shown in Figure 5A, by comparing the trauma changes in the negative control group, positive control group, and hydrogel group, it can be visualized that the use of CBM/CMC/EEO NE effectively promotes the reduction of the trauma area. By day 12, compared with the negative group, the trauma in the hydrogel treatment group no longer had blood crust, and the appearance of new skin tissue could be clearly seen. By day 16, the wounds treated with the hydrogel and positive groups almost returned to normal, while the negative group still exhibited visible scabs. Combined with results presented in Figure 5B, the wound healing rate of both the hydrogel and positive groups was higher than that of the negative treatment group throughout the treatment process. By day 12, the wound healing rate of the hydrogel treatment group reached 89.863 ± 2.608%, while that of the negative group was 82.576 ± 5.240%, showing a significant difference (*p* < 0.05). This indicates that using the CBM/CMC/EEO NE hydrogel showed more efficacy than the negative treatment group, so the EEO NE-added hydrogel significantly promotes the healing of infected wounds.

#### 3.4.2. Trauma Bacterial Load Analysis

The in vivo bacterial inhibition performance of the CBM/CMC/EEO NE hydrogel on *S. aureus* infected wounds was evaluated by measuring the change in bacterial load in trauma tissue. Figure 5C shows that using CBM/CMC/EEO NE hydrogel effectively inhibits bacterial growth in the infected trauma tissue. The bacterial load continually decreases with the duration of treatment, like the effect of antimicrobial treatment in the positive group. Combined with the results shown in Figure 5D, the bacterial load on the trauma surface of the hydrogel treatment group was already significantly lower than that of the negative group from day 4 onward. This indicates that the in vivo use of CBM/CMC/EEO NE has good inhibitory activity on the bacterial growth on infected trauma surfaces, preventing further deterioration of the trauma surface via infection, and thus facilitating trauma surface repair.

#### 3.4.3. Histological Analysis of the Trauma Surface

The histological effects of the CBM/CMC/EEO NE hydrogel on wound repair were evaluated from a histological perspective by observing and analyzing H&E and Masson’s Trichrome Staining histological sections of traumatized skin from mice.

**Figure 6:**
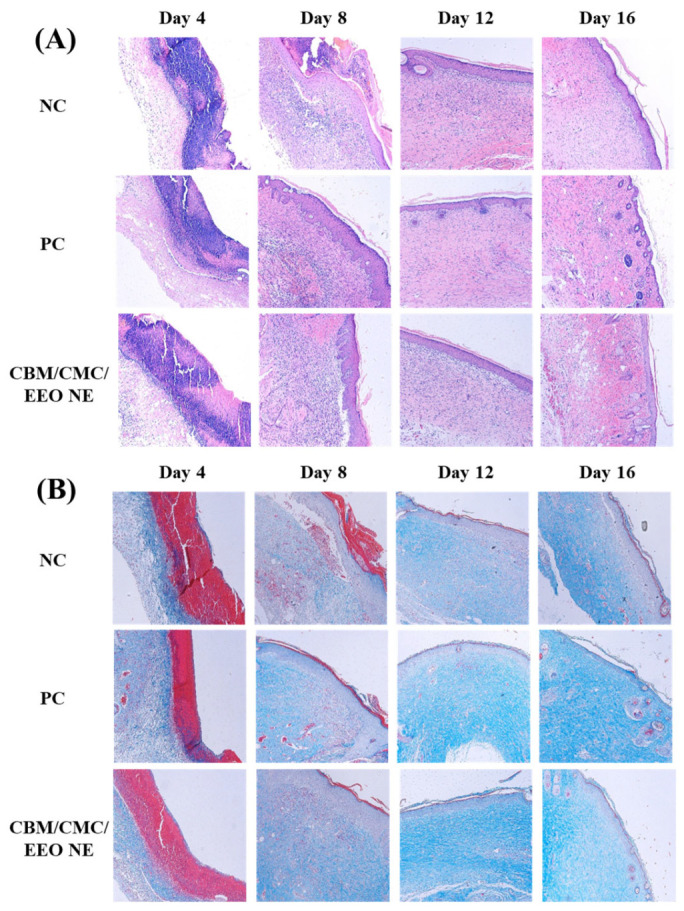
Histopathological stained sections of traumatic tissue of different groups within 16 days. (**A**) H&E stain; (**B**) Masson’s stain.

As shown in Figure 6, on the 4th day of molding the infected trauma of mice, the epidermis and dermis of all groups were destroyed, the epidermal cells were broken, and various fibrous tissues of the dermis were severely necrotic. A large amount of necrotic collagen was present, the skin appendages disappeared, and many inflammatory cells infiltrated the trauma. The inflammatory infiltration and collagen necrosis were relatively more severe in the negative group. On the 8th day, the epidermal and dermal cell tissues of the positive and hydrogel groups started to reorganize, with a large reduction in necrotic collagen, a significant appearance of new collagen, and a reduction in inflammation, while the negative group showed hyperplasia of the traumatized skin, a lack of flatness in the formation of the skin layer, and some areas with more severe inflammation and collagen necrosis remained. On the 12th day, the positive group and the gel group almost returned to normal; the skin structure was complete, the appendages began to appear, and the collagen fibers in the dermis began to arrange in an orderly manner. However, the negative group only showed initial improvement in the skin condition. There were still epidermal fractures and abnormal thickening. The skin was basically devoid of hair follicles, sweat glands, and other appendages. On the 16th day, the skin tissue of the positive group and the gel group further recovered. The epidermis and dermis cells were ordered and smooth, and hair follicles, sweat glands, and other skin accessory tissues were further formed. Additionally, the negative group still exhibited an abnormal thickening of the epidermis, and the skin accessory organs were not recovered.

The above histological analysis shows that the CBM/CMC/EEO NE hydrogel is as effective as erythromycin ointment in the recovery of *S. aureus*-infected wounds, reducing the inflammatory response, promoting the formation of new collagen, accelerating the recovery of the epidermis and dermis in a smooth and ordered manner, and regenerating skin appendages.

#### 3.4.4. Trauma Tissue Cytokine Analysis

Inflammatory and growth factors, as products secreted by lymphocytes, are important in regulating the differentiation and proliferation of inflammatory cells. They can also set immune cells to injury sites, playing an important role in tissue healing or angiogenesis. In this study, five cytokines, IL-6, TNF-α, TGF-β1, VEGF, and EGF, which have important roles in skin tissue repair, were selected as the main indicators to investigate the effects of CBM/CMC/EEO NE hydrogels on infected wounds at the cellular level.

**Figure 7:**
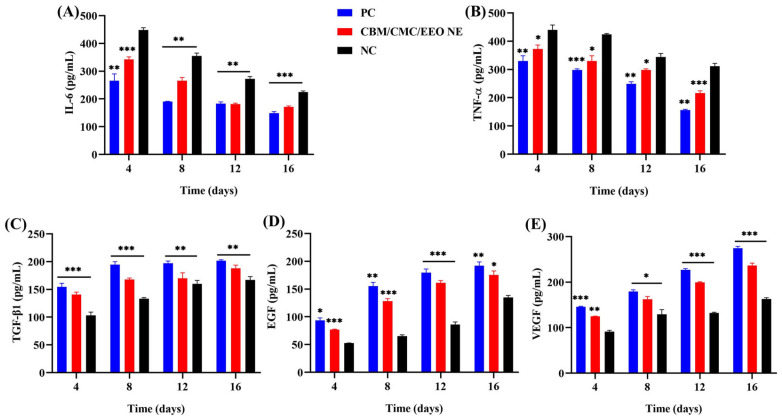
Determination (pg/mL) of IL-6 (**A**), TNF-α (**B**), TGF-β1 (**C**), EGF (**D**), and VEGF (**E**) in traumatic tissue by ELISA. (n = 3, * *p* < 0.05, ** *p* < 0.01 and *** *p* < 0.001, vs. NC).

IL-6 and TNF-α factors are related to inflammation. As shown in Figure 7A,B, IL-6 and TNF-α increased to different degrees in all treatment groups after successful molding. However, the hydrogel and positive groups had significantly lower values than the negative group. As the treatment continued, the levels of two factors, IL-6 and TNF-α, decreased continuously in the hydrogel and positive groups, being always significantly lower than in the negative group (*p* < 0.05).

TGF-β1, VEGF, and EGF cytokines are associated with cell growth and differentiation, granulation formation, and anti-inflammatory hemostasis. As shown in Figure 7C–E, mice also showed varying degrees of increase in TGF-β1, VEGF, and EGF cytokines after injury and infection. Since wounds require a high expression of these three cytokines to promote wound healing, the increase in the factor content was more significant in the hydrogel and positive groups relative to the negative group. This indicates that the use of the CBM/CMC/EEO NE hydrogel significantly promotes the expression of TGF-β1, VEGF, and EGF growth factors at the cellular level as a mean to promote rapid wound healing.

## 4. Summary

In the present study, we designed a nanoemulsion of eucalyptus essential oil with good in vitro antibacterial and anti-biofilm activity and combined it as an active substance with a hydrogel matrix physically cross-linked with Carbomer 940 and carboxymethyl chitosan to develop eucalyptus essential oil nanoemulsion hydrogels. The study results show that EEO NE has a good inhibitory and scavenging effect on the *S. aureus* biofilm. CBM/CMC/EEO NE exhibits good rheology, water retention, porosity, water vapor transmission, and biocompatibility as a trauma dressing. In vivo experiments have shown that CBM/CMC/EEO NE can effectively promote wound healing, reduce the bacterial load of wounds, and accelerate the recovery of epidermal and dermal tissue cells. The results of the trauma cytokine assay demonstrate that CBM/CMC/EEO NE significantly down-regulates the expression of two inflammatory factors, IL-6 and TNF-α, and up-regulates three growth-promoting factors, TGF-β1, VEGF, and EGF, to reduce inflammation in infected wounds while promoting wound recovery. In conclusion, CBM/CMC/EEO NE hydrogels show great potential for treating *S. aureus*-infected wounds. It is expected to be a new clinical alternative for healing infected wounds in the future.

## Data Availability

Not applicable.
